# An Immune-Associated Genomic Signature Effectively Predicts Pathologic Complete Response to Neoadjuvant Paclitaxel and Anthracycline-Based Chemotherapy in Breast Cancer

**DOI:** 10.3389/fimmu.2021.704655

**Published:** 2021-08-30

**Authors:** Changfang Fu, Yu Liu, Xinghua Han, Yueyin Pan, Hong-qiang Wang, Hongzhi Wang, Haiming Dai, Wulin Yang

**Affiliations:** ^1^Anhui Province Key Laboratory of Medical Physics and Technology, Institute of Health and Medical Technology, Hefei Institutes of Physical Science, Chinese Academy of Sciences, Hefei, China; ^2^Science Island Branch, Graduate School of University of Science and Technology of China, Hefei, China; ^3^Medical Pathology Center, Hefei Cancer Hospital, Chinese Academy of Sciences, Hefei, China; ^4^The First Affiliated Hospital of University of Science and Technology of China, Division of Life Sciences and Medicine, University of Science and Technology of China, Hefei, China; ^5^Biological Molecular Information System Laboratory, Institute of Intelligent Machines, Hefei Institutes of Physical Science, Chinese Academy of Sciences, Hefei, China

**Keywords:** chemosensitivity, breast cancer, LASSO, neoadjuvant chemotherapy, pathologic complete response

## Abstract

Breast cancer is now the leading cause of cancer morbidity and mortality among women worldwide. Paclitaxel and anthracycline-based neoadjuvant chemotherapy is widely used for the treatment of breast cancer, but its sensitivity remains difficult to predict for clinical use. In our study, a LASSO logistic regression method was applied to develop a genomic classifier for predicting pathologic complete response (pCR) to neoadjuvant chemotherapy in breast cancer. The predictive accuracy of the signature classifier was further evaluated using four other independent test sets. Also, functional enrichment analysis of genes in the signature was performed, and the correlations between the prediction score of the signature classifier and immune characteristics were explored. We found a 25-gene signature classifier through the modeling, which showed a strong ability to predict pCR to neoadjuvant chemotherapy in breast cancer. For T/FAC-based training and test sets, and a T/AC-based test set, the AUC of the signature classifier is 1.0, 0.9071, 0.9683, 0.9151, and 0.7350, respectively, indicating that it has good predictive ability for both T/FAC and T/AC schemes. The multivariate model showed that 25-gene signature was far superior to other clinical parameters as independent predictor. Functional enrichment analysis indicated that genes in the signature are mainly enriched in immune-related biological processes. The prediction score of the classifier was significantly positively correlated with the immune score. There were also significant differences in immune cell types between pCR and residual disease (RD) samples. Conclusively, we developed a 25-gene signature classifier that can effectively predict pCR to paclitaxel and anthracycline-based neoadjuvant chemotherapy in breast cancer. Our study also suggests that the immune ecosystem is actively involved in modulating clinical response to neoadjuvant chemotherapy and is beneficial to patient outcomes.

## Introduction

Breast cancer is now the leading cause of cancer incidence worldwide, with 2,261,419 new cases each year. Also, there were 684,996 deaths from breast cancer every year, making it the leading cause of death among women (1). Breast cancer is a clinically and biologically heterogeneous malignancy, with different molecular subtypes having distinct clinical features, therapy responses, and prognosis (2). Prediction of sensitivity to treatment, with the purpose to select the most effective therapy and avoiding overtreatment, is fundamental for effective precision medicine in breast cancer.

Paclitaxel and anthracycline-based neoadjuvant chemotherapy is one of the primary established treatment options for breast cancer (3); however, only about 6–30% of these patients achieved a pathologic complete response (pCR) (4). Therefore, the evaluation of sensitivity to neoadjuvant (preoperative) chemotherapy is an important task in clinical practice. Patients who achieve pCR after neoadjuvant chemotherapy have better long-term disease-free survival than patients with residual disease (RD) (5). Traditionally, histologic characteristics such as expressions of estrogen receptor (ER), progesterone receptor (PR), HER2, Ki67, and histological grade have been used as prognostic and predictive markers (6, 7). However, these general markers have limited ability in predicting individual response to treatment (7, 8), especially in patients with the same histologic characteristics and disease subtypes. On the other hand, compared to luminal subtype (2, 9), the triple-negative breast cancer (TNBC) and HER2-positive subtypes are associated with more pCR rates and higher sensitivity to neoadjuvant chemotherapy, suggesting that genetic characteristics may play an important role in chemotherapeutic sensitivity, so the characterization of gene expression profile can be used to predict treatment response and prognosis, thus guiding clinical practice.

With the development of the technologies to determine gene expressions, several studies have identified gene signatures to predict the sensitivity of individual drug (5, 8, 10). Besides, several multigene predictors were developed to predict the pCR to neoadjuvant chemotherapy and guide adjuvant therapy decisions, including Oncotype Dx to assign high, intermediate, and low recurrence score (RS) (the RS was positively correlated with the likelihood of pCR) (11), MammaPrint to define poor and good prognosis (poor prognosis was significantly associated with pCR) (12), PAM50 to classify four intrinsic subtypes of breast cancer (basal-like, HER2-enriched, and luminal B subtypes predicted pCR) (13), EndoPredict to define low and high risk (high risk was significantly associated with pCR) (14), GGI to assign high and low score (high score predicted pCR) (15), DLDA30 to predict pCR and RD (16, 17). However, most of them have limited value in predicting chemotherapy efficacy (10). A large number of genes and the small number of sampling make the model prone to overfitting and sometimes false discovery rates, leading to little clinical utility and finally unrecommended for individualized patient care (18). One of these predictors, the Oncotype Dx, which has shown predictive for chemotherapy benefit for pN0 or node-negative breast cancer patients, has been recommended by the National Comprehensive Cancer Network (NCCN) Breast Cancer Panel (3). Even though there is currently no clinically available method to predict the pCR outcome of paclitaxel and anthracycline-based chemotherapy in individual breast cancer patients, thus more robust models await development. Besides, recent studies suggest that immune responses have prognostic and predictive value for clinical outcomes and treatment responses in breast cancer (19–23), while none of the above predictors reflected immune characteristics.

The large volume of gene-expression data from many clinical studies of neoadjuvant chemotherapy provides new opportunities for the development of new predictive models. Moreover, the least absolute shrinkage and selection operator (LASSO) algorithm is better than the traditional regression method in variable selection and can reduce the correlation between independent variables to prevent overfitting of high-dimensional data (24). In this study, we adopted the powerful LASSO method to develop a new genomic predictor by using five microarray expression profile datasets in the Gene Expression Omnibus (GEO) database with patients who received neoadjuvant paclitaxel and anthracycline-based chemotherapy. Furthermore, our data suggested the clinical response to paclitaxel and anthracycline-based neoadjuvant chemotherapy is highly associated with immune ecosystem.

## Materials and Methods

### Datasets

In the GEO database (http://www.ncbi.nlm.nih.gov/geo/), we downloaded the original files (.CEL files) of microarray expression profiling datasets and the platform files, then to extract gene expression data from samples receiving paclitaxel and anthracycline-based neoadjuvant chemotherapy. A total of 744 samples were enrolled in this analysis, respectively, from GSE20271 (17), GSE25055 (5), and GSE20194 (25), which are all based on the GPL96 [HG-U133A] Affymetrix Human Genome U133A Array, GSE32646 (8) based on the GPL570 [HG-U133_Plus_2] Affymetrix Human Genome U133 Plus 2.0 Array, and GSE41998 (26) based on the GPL571 [HG-U133A_2] Affymetrix Human Genome U133A 2.0 Array. Samples in four datasets, including GSE32646, GSE20271, GSE20194, GSE25055, received paclitaxel/fluorouracil/anthracycline/cyclophosphamide (T/FAC) neoadjuvant chemotherapy. Here anthracycline refers to doxorubicin or epirubicin, which can be substituted for each other in chemotherapy regimens. In GSE32646, doxorubicin is repalced with epirubicin, while in GSE20271 and GSE20194, doxorubicin is partly replaced with epirubicin. GSE41998 dataset was derived from samples that received paclitaxel/doxorubicin/cyclophosphamide (T/AC) neoadjuvant chemotherapy. Except that GSE25055 is a HER2-negative subtype, other datasets involved all subtypes of breast cancer. Datasets enrolled different ethnic groups, including white, Asian, black, Hispanic. The workflow of this study is shown in **Figure S1**.

### Differentially Expressed Genes Analysis

Each of the original expression data underwent background correction, quantile normalization, and log2 conversion using Robust Multi-array Average (RMA) algorithm with the “affyPLM” package. The batch effect among different datasets was removed by the use of the “sva” package (27). To compare gene expression between pCR and RD samples, DEGs in GSE32646 and GSE20271 were analyzed using the “limma” package in R software. Using hierarchical clustering based on correlation distance, the differential gene results were presented as a heatmap using the R package “pheatmap.”

### Principal Component Analysis Before and After LASSO Feature Selection

The intersected DEGs between two independent datasets (GSE32646 and GSE20271) derived from different chip platforms were selected to construct the association matrix of gene expression values with pCR and RD in each dataset. Because Lasso algorithm has strong predictive power, it is used to select the optimal feature (24). The LASSO logistic regression model analysis was implemented by the “glmnet” package in R and took the non-zero regression coefficients to select the optimal biomarkers for predicting pCR and RD. Before LASSO feature selection, PCA was performed using the expression data of the intersected DEGs in all datasets. PCA was further calculated using the expression profiles of the selected genomic signature after LASSO. All PCA results were displayed in two-dimensional plots across the first two principal components.

### LASSO Logistic Regression Model Analysis

We used the LASSO method to select the optimal biomarker for predicting pCR to T/FAC neoadjuvant chemotherapy by partial likelihood deviance in minimum criteria. The group-wise classifications were calculated in 10-fold cross-validations, and area under the receiver operating characteristic (ROC) curve (AUC) was obtained by two-class logistic regression with type.measure = “auc.” As a result, LASSO method assigned a regression coefficient to each signature. Based on the results, the regression coefficients were used to construct a scoring system to weight the value of the selected signature. The formula is as follows:

$$Prediction\;Score=\sum_{i=0}^{n}\left(\beta_{\text{i}}\times x_{\text{i}}\right)$$

The “n” is the sample size. The “β” is the regression coefficient of the selected signature and is derived from the LASSO logistic regression, and “x” indicates the expression value of the selected signature. The GSE32646 dataset was used as training set. Three T/FAC-based test sets and one T/AC-based test set were used to examine the performance of the model. The scoring system was applied to predict the effect of chemotherapy. Accuracy (AC), sensitivity (SE), specificity (SP), positive predictive value (PPV), negative predictive value (NPV), and AUC were introduced to evaluate the performance of the model. The “pROC” package in R was used to draw ROC curves.

### Univariate and Multivariate Logistic Regression Analyses

To compare the independent predictive power of 25-gene signature with other clinical features (including age, tumor stage, lymph node status, histological grade, ER status, PR status, HER2 status, molecular subtype, treatment course of neoadjuvant therapy), univariate and multivariate logistic regression analyses were used to evaluate the relationship between these variables and pCR. All samples in training and test sets with complete clinical data were included in this analysis and ROC curves were plotted.

### Functional Annotations and Signaling Pathway Enrichment Analysis

“ClusterProfiler” package in R was selected for enrichment analyses of gene ontology (GO) and Kyoto Encyclopedia of Genes and Genomes (KEGG) pathway based on the selected signature. GO terms with *P* value < 0.05 were displayed. Since there is no terms with a *P* value less than 0.05, KEGG terms with a *P* value < 0.26 are shown.

### Expressive Correlations Between Genes in Signature and Immune Checkpoints

We explored the correlations of the expression values between genes in signature and immune checkpoints contained in the arrays, such as programmed cell death 1 (PD1/PDCD1), programmed cell death 1 ligand 2 (PDL2/PD1L2), cytotoxic T-lymphocyte antigen 4 (CTLA4), lymphocyte activation gene 3 protein (LAG3), Indoleamine 2,3-dioxygenase 1 (IDO1), etc.

### ESTIMATE Analysis

Stromal and immune cells are the major fraction in the tumor microenvironment and have an important role in tumor biology. ESTIMATE algorithm was performed to measure the abundance of immune and stromal cells in each sample using expression data of training and test sets. Thus, stromal score and immune score were calculated, and tumor purity of each sample was inferred with “ESTIMATE” package in R (28). The difference in the abundance of immune and stromal cells between pCR and RD samples in all datasets was analyzed. The correlations between the prediction score and the stromal score, immune score, ESTIMATE score, tumor purity were further explored.

### CIBERSORT Analysis

CIBERSORT is an established deconvolution algorithm that uses a leukocyte signature matrix (LM22) that contained 547 reference genes to infer the relative proportions of 22 human hematopoietic cells including T cells, B cells, plasma cells, NK cells, and myeloid subsets (23, 29). We use CIBERSORT to evaluate the tumor-infiltrating immune cells for each sample. Differential immune cell type fractions were analyzed between pCR and RD samples in all datasets.

### Correlation Between Genes in Signature and Immune Cell Infiltrates

The genes in signature were rank-ordered based on the magnitude of the coefficient in the LASSO model. The top genes were applied to explore their correlations with immune score and compare the differential immune cell types between pCR and RD samples. Prognostic significances of the top genes in breast cancer were analyzed based on the Kaplan-Meier (KM) plotter database (http://kmplot.com).

### Statistics Analysis

Statistical analysis was performed in R (version 3.6.1; https://www.R-project.org). DEGs between pCR and RD samples were analyzed using unpaired t-tests provided by “limma” package. LASSO logistic regression analysis was performed to construct the model. Wilcoxon rank sum test was used for differences between two groups, and Kruskal-Wallis test was used for differences between more than two groups. Correlations between genes in signature and immune checkpoints or immune cell infiltrates were evaluated by the Pearson correlation coefficient. *P* value < 0.05 was considered statistically significant.

## Results

### DEGs and Hierarchical Clustering

Before modeling, a training data set and four test sets of breast cancer patients were collected from GEO database. The clinicopathological characteristics of the training set and test sets are shown in **Table 1**. First, 238 DEGs and 224 DEGs were identified between pCR and RD samples from the training dataset GSE32646 and the testing dataset GSE20271, respectively, and then filtered by the criteria of adjusted *P* value < 0.05, |log_2_ FC| > 0.6. This analysis resulted in an intersecting part that consists of 54 genes between the two groups of DEGs. The expression matrices of these 54 genes in GSE32646 were then selected for the modeling of the training set, and the expression matrices of these 54 genes in GSE20271, GSE20194, GSE25055, and GSE41998 were selected as the test sets, respectively. Furthermore, the expression matrix of 25 genes in the training set GSE32646 was analyzed and displayed by heat map (**Figure S2**). The hierarchical clustering results based on the heat map showed that the expression patterns of these 25 genes could preliminarily distinguish pCR samples from RD samples.

**Table 1:** Clinical characteristics of patients.

Characteristic	GSE32646 (n = 115)	GSE20271 (n = 74)	GSE20194 (n = 207)	GSE25055 (n = 227)	GSE41998 (n = 121)
Age (years)					
<65	102	61	172	197	113
≥65	13	13	35	30	8
Tumor stage					
T0	0	1	3	2	0
T1	5	3	20	19	2
T2	87	34	119	131	79
T3	18	18	31	35	40
T4	5	18	34	40	0
Lymph node status					
Positive	83	46	139	165	NA
Negative	32	28	68	62	NA
Histological grade					
1	16	9	10	13	NA
2	78	25	82	92	NA
3	21	27	115	122	NA
NA		13			
ER status					
Positive	71	41	125	131	45
Negative	44	33	82	96	76
PR status					
Positive	45	36	90	102	46
Negative	70	38	117	125	75
HER2 status					
Positive	34	14	45	0	9
Negative	81	60	162	227	112
Neoadjuvant therapy					
Weekly T×12+FAC×4	115	74	119	227	0
3-weekly T×4+FAC×4	0	0	88	0	0
Weekly T×12+AC×4	0	0	0	0	121
Pathologic response					
RD	88	57	161	184	87
pCR	27	17	46	43	34

ER, Estrogen Receptor; PR, Progesterone Receptor; HER2, Human Epidermal Growth Factor Receptor 2; RD, Residual Disease; pCR, Pathologic Complete Response; NA, Not Available.

### PCA and Feature Selection Using the LASSO Method

**Figure 1:**
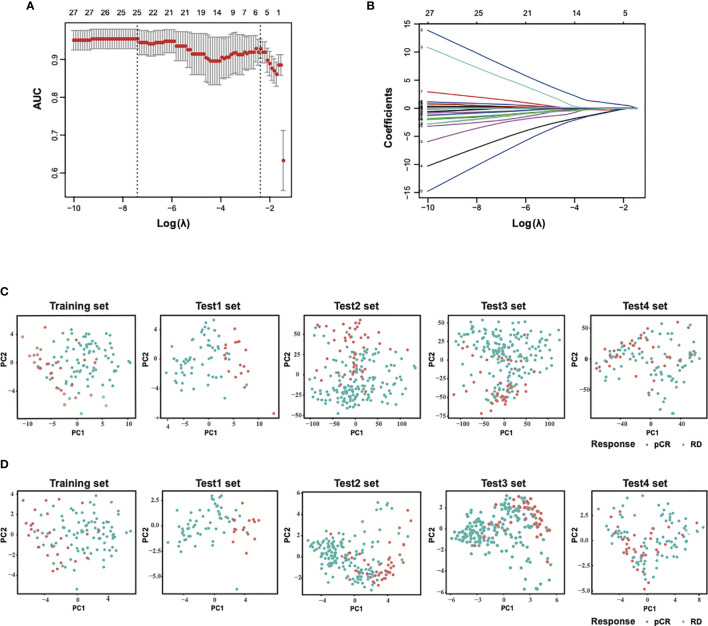
Construction of LASSO model and principal component analysis. **(A)** Ten-fold cross-validation for tuning parameter selection in the LASSO model. **(B)** LASSO coefficient profiles of the training set. **(C)** PCA before and **(D)** after LASSO feature selection in the training set and each test sets. LASSO, least absolute shrinkage and selection operator; PCA, principal component analysis. Training set, GSE32646; Test1 set, GSE20271; Test2 set, GSE20194; Test3 set, GSE25055; Test4 set, GSE41998.

To build the model to predict sensitivity to paclitaxel and anthracycline-based chemotherapy in breast cancer, the patients in the training dataset were grouped into pCR or RD groups based on the patient response status, and the expression values of the selected 54 genes from GSE32646 were extracted and analyzed by LASSO regression model analysis. A 25-gene signature classifier was then identified with non-zero regression coefficients as optimal biomarkers (**Figures 1A** **, B**). The 25 genes were ADAMDEC1, CCL18, CD79A, CD96, CXCL13, DIRAS3, ERBB4, EVL, GAMT, GBP1, GFRA1, GZMB, HSPB8, IGHM, IRS1, ITK, LOC102723479, MAPT, PADI2, RLN2, SEL1L3, SERPINA5, STC2, STK32B, SYBU. PCA results of 54 genes before LASSO selection and 25 genes after LASSO selection were presented for the training set and test sets, respectively (**Figures 1C** **, D**). These results demonstrated that samples that differed in response to paclitaxel and anthracycline-based neoadjuvant chemotherapy were more easily distinguished by the 25-gene signature classifier.

### The LASSO Logistic Regression Model Construction

As mentioned above, using the LASSO method, a 25-gene signature was identified as the optimal feature and the value of lambda.min = 0.000798599. The prediction score of the signature classifier is calculated using the following formula:

Prediction Score = ADAMDEC1×0.00321747620626765 + CCL18×0.0457079749167309 + CD79A×8.61152358256599 + CD96×6.22205851428899 + CXCL13×(−0.585126092824241) + DIRAS3×(−6.08198493202845) + ERBB4×1.72908010036751 + EVL×(−1.70368931131805) + GAMT×(−8.84896004120253) + GBP1×(−0.764626193845283) + GFRA1×(−0.115908259316488) + GZMB×(−0.0752619689246736) + HSPB8×(−1.28866942797256) + IGHM×(−1.37319937849059) + IRS1×0.250096649476748 + ITK×(−2.30297033083433) + LOC102723479×0.385454564188641 + MAPT×0.286187494306212 + PADI2×0.783128470665541 + RLN2×(−1.56204367828805) + SEL1L3×(−2.98426861278556) + SERPINA5×0.25651424658033 + STC2×0.430345120497431 + STK32B×(−1.28399430856461) + SYBU × (−0.706271090221699)

**Figure 2:**
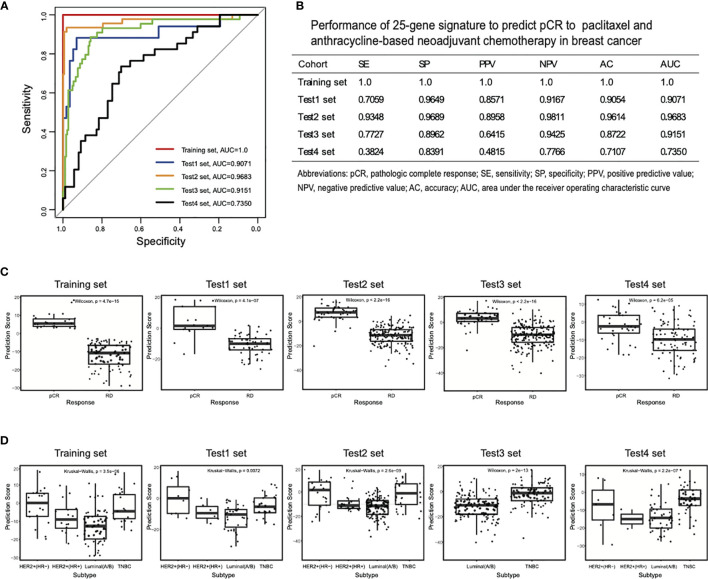
Receiver operating characteristic (ROC) curves for the 25-gene signature classifier and comparison of prediction scores between pCR and RD samples. **(A)** ROC analyses in the training set and four test sets. **(B)** The performance parameters of the signature classifier in pCR prediction. **(C)** The prediction scores of the classifier were higher in pCR than RD samples in all training and test sets. **(D)** The prediction scores of HER2+ (HR−) and TNBC subtypes were higher than those of other subtypes. pCR, pathologic complete response; RD, residual disease.

As shown in **Figures 2A, B**, the AUC and AC values of the model were 1 and 1, respectively, for the T/FAC-based training dataset. We further evaluated with this signature in the two other independent T/FAC-based test sets, and the AUC and AC values were 0.9071, 0.9683 and 0.9054, 0.9614, respectively. More importantly, all of the above training and testing sets cover all of the subtypes of breast cancer, suggesting the predictive accuracy independent of cancer subtypes for our model. Moreover, the AUC and AC values of the signature classifier were 0.9151 and 0.8722, respectively, in the T/FAC-based test set, which included only the HER2-negative subtype. Further analysis indicated the model also showed a high discrimination ability with the SE of 1, 0.7059, 0.9348, 0.7727; the SP of 1, 0.9649, 0.9689, 0.8962; the PPV of 1, 0.8571, 0.8958, 0.6415; the NPV of 1, 0.9167, 0.9811, 0.9425 for the T/FAC-based training set and three test sets. Importantly, the model also performed well on another T/AC-based test set involved all types of breast cancer, in which the AUC, AC, SE, SP, PPV, NPV was 0.7350, 0.7107, 0.3824, 0.8391, 0.4815, 0.7766, respectively. Collectively, the 25-gene signature classifier showed excellent predictive performances on both T/FAC and T/AC neoadjuvant chemotherapy regardless of tumor subtype, although it did better in predicting T/FAC response.

Further, we compared the prediction scores of the 25-gene signature classifier between pCR and RD samples or different subtypes, and the results showed that it could distinguish the two groups of samples well, both in the training and test sets (**Figure 2C**) and between HER2-positive (HR-negative), HER2-positive (HR-positive), Luminal (A/B), and TNBC subtypes of breast cancer (**Figure 2D**). In general, the prediction scores were higher in pCR samples than RD samples in all datasets. In four molecular subtypes, HER2-positive (HR-negative) and TNBC subtype showed higher prediction scores than HER2-positive (HR-positive) and Luminal (A/B) subtype, implying different cancer subtypes response differently to chemotherapy, which was consistent with previous studies (2, 5, 9).

### Independent Predictive Role of the 25-Gene Signature

**Figure 3:**
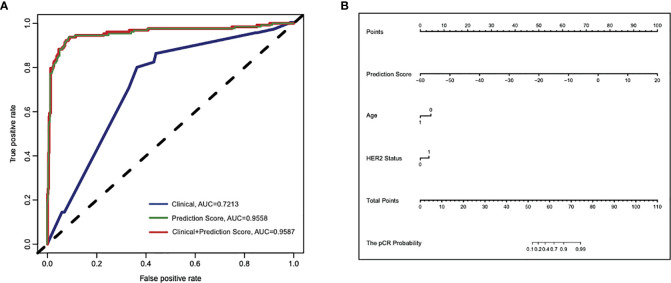
Cross-validated ROC curves based on the multivariate logistic regression model for predicting pCR to paclitaxel and anthracycline-based neoadjuvant chemotherapy in breast cancer. **(A)** The area under ROC curve (AUC) of the 25-gene signature alone or in combination with clinical features were 0.9558 and 0.9587, respectively, compared with the lower AUC (0.7213) of the model that only included clinical features. **(B)** A constructed nomogram for predicting pCR to paclitaxel and anthracycline-based neoadjuvant chemotherapy in breast cancer. pCR, pathologic complete response.

In order to evaluate how well the clinical characteristics alone or in combination with the 25-gene signature classifier predict pCR to paclitaxel and anthracycline-based neoadjuvant chemotherapy, univariate and multivariate logistic regression analyses were performed (**Table S1** and **Table 2**). Although univariate analysis showed that several clinical features were significantly associated with pCR, multivariate analysis showed that only variables such as age and HER2 status were independent predictors in addition to the 25-gene signature classifier. The ROC curves showed that the AUC based on the 25-gene signature was 0.9558, and the AUC after being combined with clinical covariates increased slightly to 0.9587 (**Figure 3A**), indicating the 25-gene signature classifier plays a leading role in pCR prediction. In addition, a nomogram was constructed based on independent prognostic factors derived from the multivariate analysis (**Figure 3B**), which also showed that 25-gene signature had a dominant contribution to pCR prediction relative to other clinical variables.

**Table 2:** Multivariate logistic regression analyses for predicting pCR to neoadjuvant chemotherapy.

Intercept and variable	Analysis without 25-gene signature			Analysis with 25-gene signature		
	β	OR (95% CI)	*P* value	β	OR (95% CI)	*P* value
Intercept	−2.559	0.077 (0.006–1.016)	0.046	0.332	1.393 (0.024–104.417)	0.876
Age	−0.884	0.413 (0.188–0.830)	0.018	−1.332	0.264 (0.066–0.882)	0.043
Lymph node status	0.352	1.422 (0.845–2.444)	0.192	−0.240	0.786 (0.345–1.805)	0.567
Histological grade	0.470	1.600 (1.035–2.514)	0.037	0.349	1.418 (0.721–2.848)	0.317
ER status	−1.309	0.270 (0.052–1.217)	0.1	−1.305	0.271 (0.017–3.699)	0.341
PR status	−0.264	0.768 (0.401–1.472)	0.424	−0.188	0.829 (0.341–2.010)	0.676
HER2 status	0.934	2.545 (1.408–4.459)	0.001	1.001	2.722 (1.042–6.991)	0.038
Molecular subtype	0.148	1.159 (0.637–2.035)	0.615	−0.371	0.690 (0.258–1.740)	0.444
25-gene signature				0.364	1.439 (1.345–1.557)	<0.001

ER, Estrogen Receptor; PR, Progesterone Receptor; HER2, Human Epidermal Growth Factor Receptor 2; OR, Odds ratio.

### Enrichment Analysis of the 25 Genes in the Signature

**Figure 4:**
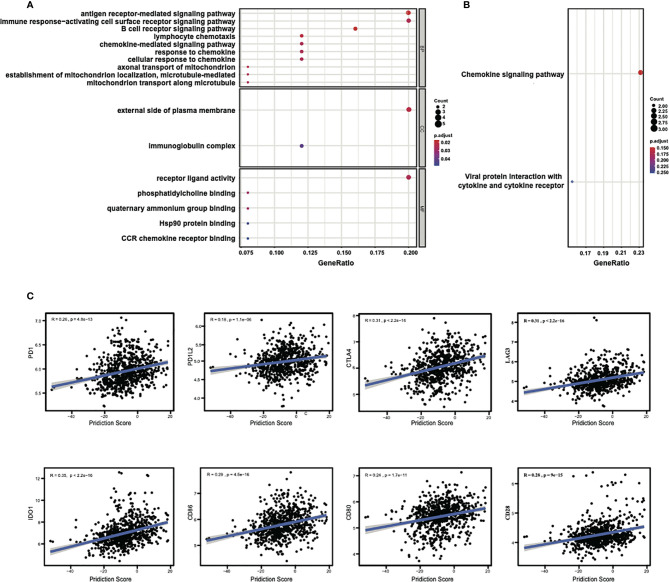
Enrichment analysis of the 25 genes in signature and the correlations of the prediction score with the expressions of immune checkpoints. **(A)** Significantly enriched GO terms and **(B)** significantly enriched immune-related KEGG pathways. LOC102723479 is replaced with official gene symbol of IGHG1 by querying in the NetAffx Analysis Center and verified by BLAST search in NCBI database using probe sequence. **(C)** The prediction scores of the classifier show significant positive correlations with the expression of the immune checkpoints, especially with CTLA4, LAG3, and IDO1.

The above results indicated a good performance of the 25-gene signature classifier in the prediction of chemotherapy responses in breast cancer and suggested that the genes contained in the signature may play an important role in influencing the response to chemotherapy. To analyze the potential molecular pathways affecting the response, GO and KEGG enrichment analyses were performed to examine the potential cellular processes or pathways related to 25 genes in signature. We found many of these 25 genes are dominantly involved in immune response processes/pathways. The significantly enriched GO terms in the biological process (BP) includes “B cell receptor signaling pathway,” “antigen receptor-mediated signaling pathway,” “lymphocyte chemotaxis,” “axonal transport of mitochondrion,” “chemokine-mediated signaling pathway,” etc. (**Figure 4A**). The enriched GO terms in cellular component (CC) include “external side of plasma membrane,” while in molecular function (MF) include “receptor ligand activity.” KEGG enrichment results indicated that these genes were related to “chemokine signaling pathway” (**Figure 4B**). In summary, the GO enrichment results suggest that membrane receptor-mediated immune signaling cascade may play a predominant role in the sensitivity to neoadjuvant chemotherapy in breast cancer.

Because of the involvement of immune response pathways, we explored the relationships of 25-gene signature with immune checkpoints. The correlation between the prediction scores of the signature classifier and the mRNA expressions of immune checkpoints (PD1, PD1L2, CTLA4, etc.) for all samples in the training and test sets were estimated. Through this analysis, a significant correlation was found between the prediction score and the expression of different immune checkpoints, with the Pearson coefficients: with PD1 (R = 0.26, *P* = 4.8e-13), with PD1L2 (R = 0.18, *P* = 1.1e-06), with CTLA4 (R = 0.31, *P* < 2.2e-16), with LAG3 (R = 0.31, *P* < 2.2e-16), with IDO1 (R = 0.35, *P* < 2.2e-16), with CD86 (R = 0.29, *P* = 4.5e-16), with CD80 (R = 0.24, *P* = 1.7e-11), with CD28 (R = 0.28, *P* = 9e-15), as shown in **Figure 4C**. There were also consistent results across other immune checkpoints (**Table S2**). These results again suggest that immune regulation might play a key role in the prognosis of breast cancer chemotherapy.

### Differential Immune Cell Abundances Between pCR and RD Samples

**Figure 5:**
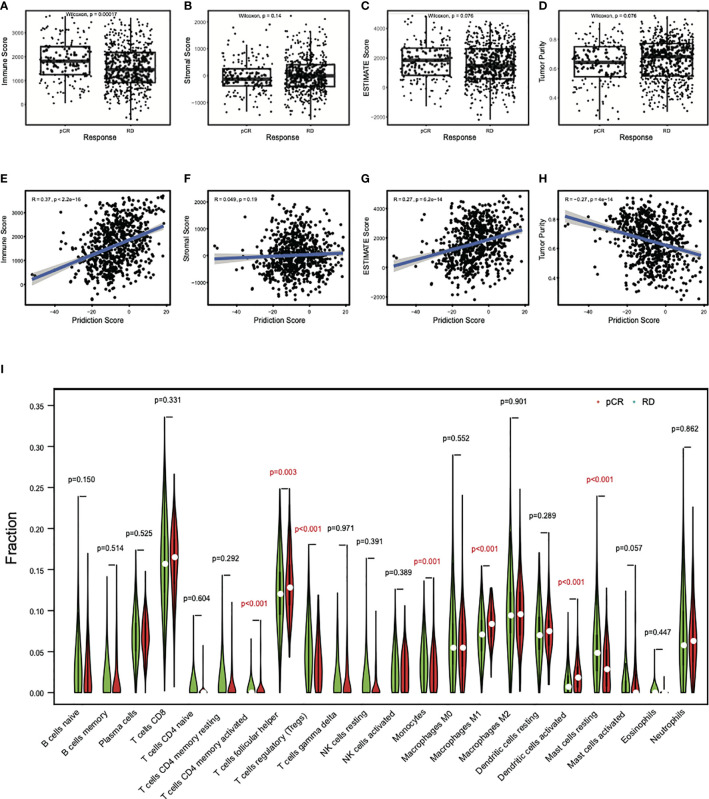
Differential immune cell abundances between pCR and RD samples. **(A)** The mean immune score in pCR samples was significantly higher than that in RD samples. **(B)** The mean stromal score showed no difference between pCR and RD samples. Compared to RD samples, **(C)** the mean ESTIMATE score was higher in pCR samples, but **(D)** tumor purity decreased. Prediction scores were positively correlated with **(E)** immune scores and **(G)** ESTIMATE scores, but not with **(F)** stromal scores. **(H)** There was a significant negative correlation between prediction score and tumor purity. **(I)** Immune cell types showed significant difference between pCR and RD samples. The fractions of “T cells CD4 memory activated,” “T cells follicular helper,” “Macrophages M1,” and “Dendritic cells activated” were significantly higher in pCR than RD samples, whereas the fractions of “T cells regulatory,” “Monocytes,” and “Mast cells resting” were significantly lower in pCR than RD samples. pCR, pathologic complete response; RD, residual disease.

To further investigate the relationship of the clinical response with the involvement of immune cells in these breast cancer patients, we analyzed the abundance of immune and stromal cells in all datasets by the ESTIMATE algorithm. The results showed that the immune scores in pCR samples were significantly higher than that in RD samples (**Figure 5A**), while no differences were observed for the stromal scores, indicating that the immune cells in the pCR samples were relatively enriched (**Figure 5B**). Consistently, ESTIMATE scores were higher in the pCR samples (**Figure 5C**). But tumor purities in the pCR samples decreased (*P* = 0.076) (**Figure 5D**), indicating that pCR samples contained diverse cell types. Consistent with this observation, there were significant positive correlations between the prediction scores and immune scores (R = 0.37, *P* < 2.2e−16) (**Figure 5E**), and between the prediction scores and ESTIMATE scores (R= 0.27, *P* = 6.2e−14) (**Figure 5G**), but a significant negative correlation between the prediction scores and tumor purities (R = − 0.27, *P* = 4e−14) (**Figure 5H**). There was no correlation between prediction scores and stromal scores (R = 0.049, *P* = 0.19) (**Figure 5F**). These results suggest that the immune microenvironment may contribute to the sensitivity of breast cancer to chemotherapy.

To obtain which types of immune cells might be involved, we studied the difference in the immune cell content between pCR and RD samples by CIBERSORT analysis. T cells CD8 and T cells follicular helper (Tfh) were the two most common infiltrating immune cells in pCR and RD samples of all datasets. Their contents were both increased in pCR samples, and the increased content of Tfh was significant (*P* = 0.003) (**Figure 5I**). In total, seven differential immune cell types, including “T cells CD4 memory activated,” “T cells regulatory (Tregs),” “Tfh,” “Monocytes,” “Macrophages M1,” “Dendritic cells activated,” and “Mast cells resting” showed a significant difference between pCR and RD samples. The fractions of “T cells CD4 memory activated,” “Tfh,” “Macrophages M1,” and “Dendritic cells activated” were significantly higher in pCR than RD samples, whereas the fractions of “Tregs,” “Monocytes,” and “Mast cells resting” were significantly lower in pCR samples than in RD samples. These immune cells interact with the process of chemotherapy, and the variety and number of these cells may lead to the difference in the outcome of chemotherapy.

### CD79A and CD96 Were Highly Related With Immunity and Predicted a Better Prognosis

**Figure 6:**
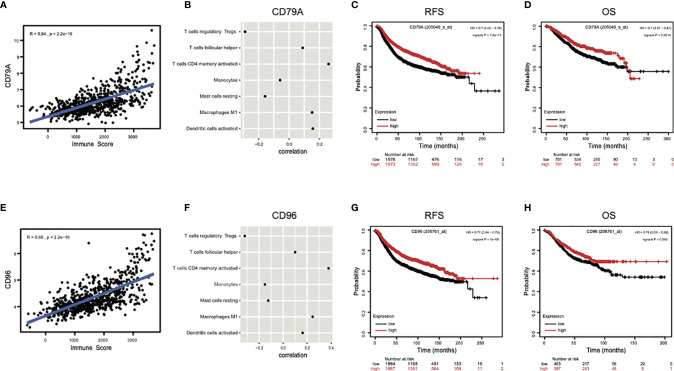
The genes contained in the signature reflect immune response and prognosis. **(A, E)** The expressions of two genes with highest coefficient, CD79A and CD96, presented strong positive correlations with immune scores. **(B, F)** The expressions of CD79A and CD96 also showed significant positive correlations with immune infiltrations of “T cells CD4 memory activated,” “T cells follicular helper,” “Macrophages M1,” “Dendritic cells activated,” and significant negative correlations with “T cells regulatory,” “Monocytes,” and “Mast cells resting.” **(C, D, G, H)** The increased expressions of CD79A and CD96 were significantly associated with higher RFS, OS in breast cancer by Kapan-Meier survival analysis. RFS, relapse-free survival; OS, overall survival; HR, hazard ratio.

Next, to explore the association between specific genes in signature and immunity, the two genes that showed the highest coefficients, CD79A and CD96, were selected for correlation analysis of its expressions with immune scores or abundance of immune infiltrations. Results indicated the expressions of both CD79A (R = 0.64, *P* < 2.2e−16) (**Figure 6A**) and CD96 (R = 0.68, *P* < 2.2e−16) (**Figure 6E**) showed a strong positive correlation with immune scores. Their expressions also showed significant positive correlation with the infiltrations of “T cells CD4 memory activated,” “Tfh, Macrophages M1,” “Dendritic cells activated,” but a significant negative correlation with “Tregs,” “Monocytes,” “Mast cells resting” (**Figures 6B, F**). Survival analysis indicated the increased expression of CD79A and CD96 was significantly associated with favorable relapse-free survival (RFS), overall survival (OS) in breast cancer (**Figures 6C, D** **, G, H**). These results suggest that high expression of CD79A and CD96 in pCR samples can reflect a high level of immune responses, which is beneficial to patient survival. Collectively, the immune ecosystem may play an important role in the sensitivity of breast cancer patients to chemotherapy and is closely related to their prognosis.

## Discussion

In this study, we developed an immune-associated 25-gene signature classifier by LASSO logistic method to predict pCR to paclitaxel and anthracycline-based neoadjuvant chemotherapy from five datasets received T/FAC and T/AC regimens in breast cancer. The genomic signature was firstly derived from the T/FAC-based training set. The predictive power of this signature classifier was confirmed by four independent test sets across different platforms, including three T/FAC sets and one T/AC set, and demonstrated an excellent predictive accuracy. Previous studies have reported multigene predictors to predict responses in cancer patients, such as Oncotype Dx, MammaPrint, PAM50, EndoPredict, GGI, and DLDA30. However, only PAM50, GGI, and DLDA30 were purely established on the T/FAC regimen. Moreover, these predictive models did not achieve effective prediction abilities, for example, the AUC of the PAM50 subtype Cox model was 0.781 (13), the AUC of the multivariate logistic regression model combining GGI and clinical covariates was 0.7350 (15), and DLDA30 using Diagonal Linear Discriminant Analysis achieved an AUC of 0.877, but got only 0.711 when evaluated in a clinical trial (16, 17). The PPV did not show better results, with that of the PAM50, GGI, and DLDA30 were 0.432 (13), 0.404 (15), 0.520 (16), respectively, and DLDA30 had a PPV of 0.380 when re-evaluated (17). Compared with those predictors, our model achieved far better performance. This prediction model may be suitable for both T/FAC and T/AC schemes, although a slightly higher AUC, AC, SE, and PPV and better SP and NPV in T/FAC-based datasets were observed than those in the T/AC-based dataset. This may be because the training set is based on the T/FAC chemotherapy regimen. Although the drug composition of the T/AC regimen is slightly different, the 25-gene signature classifier appears to be able to provide effective predictive accuracy for the T/AC regimen as well. In the future, gene expression data based on the T/AC chemotherapy can be collected as a training set to construct a classifier, to improve the prediction accuracy of T/AC treatment response. Importantly, GSE25055 only contains data for HER2-negative subtypes, and the model’s good predictive performance for this group of patients suggests that this classifier can be applied effectively to HER2-negative breast cancer. Moreover, because genetic data in this study comes from multi-ethnic populations, it can be applied to different ethnic groups. Collectively, based on AUC, AC, SE, SP, PPV, and NPV values, the model shows good predictive ability, reproducibility, and generalizability over different platforms of datasets and different subtypes of breast cancer, and has good clinical application potential.

Traditional clinical and pathological predictors such as ER, PR, HER2, histological grade, or Ki-67 cannot provide individualized treatment strategies for breast cancer and often cause overtreatment with chemotherapy (10). In the current study, we used multivariate logistic regression to evaluate the independent predictive ability of 25-gene signature and other clinical variables for pCR, showing the significant advantage of 25-gene signature in pCR prediction. Therefore, the 25-gene signature classifier has the promise to be an effective tool for predicting neoadjuvant chemotherapy sensitivity in breast cancer. In multigene assays developed to predict chemotherapy response, only Oncotype Dx is recommended by the NCCN Guideline for breast Cancer to guide clinical decision-making. The Oncotype Dx panel includes 16 cancer-related genes involved in the proliferation and invasion, HER2, Estrogen, and other five control genes (30) and 25 genes in our classifier are dominantly immune-related. Differences in their genetic makeup might account for their different predictive powers. The GO and KEGG enrichment analyses showed most of the 25 genes in the signature are involved in various immune-related biological processes/pathways, such as “B cell receptor signaling pathway,” “antigen receptor-mediated signaling pathway,” “immune response-activating cell surface receptor signaling pathway,” “receptor ligand activity,” implying that membrane receptor-mediated immune signaling cascade is one of the key determinants of the sensitivity to paclitaxel and anthracycline-based neoadjuvant chemotherapy in breast cancer. We further investigated the relationships of 25-gene signature with immune checkpoints, some of which have been widely used in tumor immunotherapy. The results showed that the predictive scores had a significant positive correlation with the immune checkpoints, such as PD1, PD1L2, and was particularly highly correlated with CTLA4, LAG3, and IDO1, suggesting immune regulation plays a key role in the prognosis of breast cancer chemotherapy (31, 32).

As we know, breast cancer is not classically considered immunogenic (33, 34). Recent insights into the association between immune microenvironment and risk of recurrence have revealed that the tumor-infiltrating lymphocytes (TILs) were closely related to the prognosis of breast cancer (31, 35, 36). Clinical data suggested TILs collaborate with the action of chemotherapy and contribute to pCR to neoadjuvant treatment in breast cancer (37, 38). High TILs are strongly associated with improved disease-free survival (DFS), distant recurrence-free interval (DRFI), and OS, suggesting that host antitumor immune engagement may be a key survival determinant (39–41). Previous studies showed the stromal TILs were 10% in ER-positive and HER2-negative subtype, 15% in HER2-positive subtype, and 20% in ER-negative and HER2-negative subtype of breast cancer (42). The immune score can directly reflect the degree of immune infiltration in tumor tissue. In our study, the immune score was significantly higher in pCR than RD samples and had a significant positive correlation with the prediction score. It has been reported that TILs are a predictor for an increased pCR rate after paclitaxel and anthracycline-based neoadjuvant chemotherapy in HER2-positive and TNBC (5, 37, 38, 43). The current study showed that the HER2-positive (HR-negative) and TNBC subtypes have higher prediction scores than the HER2-positive (HR-positive) and Luminal (A/B) subtypes, suggesting that the rate of pCR to neoadjuvant T/FAC chemotherapy in breast cancer was correlated with luminal status. Thus, our data confirm previous studies that HER2-positive (HR-negative) and TNBC subtypes of breast cancer are more sensitive to paclitaxel and anthracycline-based neoadjuvant chemotherapy than HER2-positive (HR-positive) and Luminal (A/B) subtypes of breast cancer. Our data also verified that the immune system engaged in clinical response to chemotherapy. However, underlying mechanisms of TILs in response to chemotherapy in different subtypes of breast cancer are still largely unknown.

TILs in breast cancer mainly comprise CD8 T cells, CD4 T cells, B cells, dendritic cells, and granulocytes (19, 44). CD4 T cells contained different lineages, namely, Th1, Th2, Th17, Tfh, FoxP3+ Tregs (44). Based on our analysis, CD8 T cells and Tfh were the two most common immune cells in breast cancer. Our work showed that TILs in pCR samples contained more T cells CD4 memory activated, Tfh, macrophages M1, dendritic cells activated and lower Tregs, monocytes, mast cells resting than that in RD samples. Previous studies suggested Tregs can suppress CD8 T cell cytotoxicity, inhibit adaptive antitumor immunity, and promote tumor metastasis (19, 23). High ratios of CD8/Tregs were found to correlate with high pCR to neoadjuvant chemotherapy. Dendritic cell activated can trigger CD8 T cell antitumor immunity and prime the differentiation process from naive CD4 T cell to Tfh cell (45). PD1+ Tfh cell can produce CXCL13 and macrophages M1 present antigen to T cells (46). The difference in the number of these seven types of immune cells identified by our work may be the cause of the changes in immune regulation, which interact with the chemotherapy process, and maybe the internal cause of the predictive model’s effect. On the other hand, the expression levels of the genes included in the model can also reflect levels of immune cell infiltration. We studied the two genes with the highest signature coefficients, CD79A and CD96, and found that they were significantly associated with immune scores and the infiltrations of different immune cell types and contributed to better prognostic survival in breast cancer patients. These genes may act as a marker of immune cells or directly regulate the immune-related antitumor process in response to chemotherapy. Recent studies have indicated certain chemotherapy drugs induced immunogenic cell death, while some other chemotherapy drugs did not (47). For example, anthracycline has been reported to induce immunogenic cell death in colon cancer cells and mouse models (48). Further studies also suggested anthracycline-induced immunogenic cell death in breast cancer and fibrosarcoma cells and mouse models (49, 50). While these breast cancer patients have been treated with an anthracycline-based regimen, the induction of immunogenic cell death by anthracycline in these patients might be the reason that many of the immune response genes were involved. The specific molecular mechanisms in the regulation of chemosensitivity in breast cancer are still to be explored in the future.

Our investigation has several limitations. First, our study is retrospective. Second, we looked at all types of breast cancer and did not focus on specific subtypes due to limited samples. Although our model also shows good predictive ability in predicting specific isoforms such as HER2 negative subtype, different isoforms may still have different molecular bases that need to be reflected by more sophisticated models. Third, the training set of our model is constructed based on the T/FAC regimen, which has limited predictive ability for the T/AC regimen widely used in clinical practice. It is still waiting for the future to obtain and use more T/AC scheme datasets to build the model and verify the prediction validity.

In conclusion, we developed a predictive model with a 25-gene signature for prediction pCR to paclitaxel and anthracycline-based neoadjuvant chemotherapy in breast cancer regardless of subtypes and indicated an involvement of the immune regulations in chemotherapy sensitivity at the genetic level. It is thought that the classification of patients based on the immune ecosystem will facilitate the implementation of precision medicine approaches (51). Therefore, in the selection of neoadjuvant chemotherapy regimens for breast cancer and the design of accurate treatment regimens, priority should be given to polygenic tests that reflect patient immunomodulation. The 25-gene signature classifier presented in this study, reflecting host immune regulations, might provide a practical approach to the precise treatment of breast cancer patients.

## Data Availability Statement

The original contributions presented in the study are included in the article/**Supplementary Material**. Further inquiries can be directed to the corresponding authors.

## Author Contributions

Conception and design: WY, CF, and HD. Development of methodology: WY, CF, HD, and YL. Acquisition of data: HD, XH, YP, H-QW, and HW. Analysis, validation, and interpretation of data: WY, CF, HD, YL, XH, YP, H-QW, and HW. Writing, review, and/or revision of the manuscript: CF, WY, HD, YL, XH, YP, H-QW, and HW. Administrative, technical, or material support: XH, YP, H-QW, and HW. Study supervision: WY and HD. All authors contributed to the article and approved the submitted version.

## Funding

This study was in part supported by the National Natural Science Foundation of China (81872276, 61973295), the foundation of Anhui Province Key Laboratory of Medical Physics and Technology (LMPT201906, LMPT201907, LMPT201908), and the Anhui Province’s Key Research and Development Project (201904a07020092).

## Conflict of Interest

The authors have applied for a Chinese patent (patent No. 202110320031.5) for genomic signature, a method for predicting neoadjuvant chemotherapy sensitivity proposed in this study. The patent is owned by the Hefei Institutes of Physical Science, Chinese Academy of Sciences.

The authors declare that the research was conducted in the absence of any commercial or financial relationships that could be construed as a potential conflict of interest.

## Publisher’s Note

All claims expressed in this article are solely those of the authors and do not necessarily represent those of their affiliated organizations, or those of the publisher, the editors and the reviewers. Any product that may be evaluated in this article, or claim that may be made by its manufacturer, is not guaranteed or endorsed by the publisher.

## Supplementary Material

Supplementary Figure 1 The workflow of this study.

Supplementary Figure 2 Hierarchical clustering dendrograms of the expression patterns of 25 DEGs in all datasets. DEGs, differentially expressed genes.
