# Supplementary figures and images for: An Immune-Associated Genomic Signature Effectively Predicts Pathologic Complete Response to Neoadjuvant Paclitaxel and Anthracycline-Based Chemotherapy in Breast Cancer
Source: Front Immunol. 2021 Aug 30;12:704655. doi: 10.3389/fimmu.2021.704655 (PMC8435784; doi:10.3389/fimmu.2021.704655)

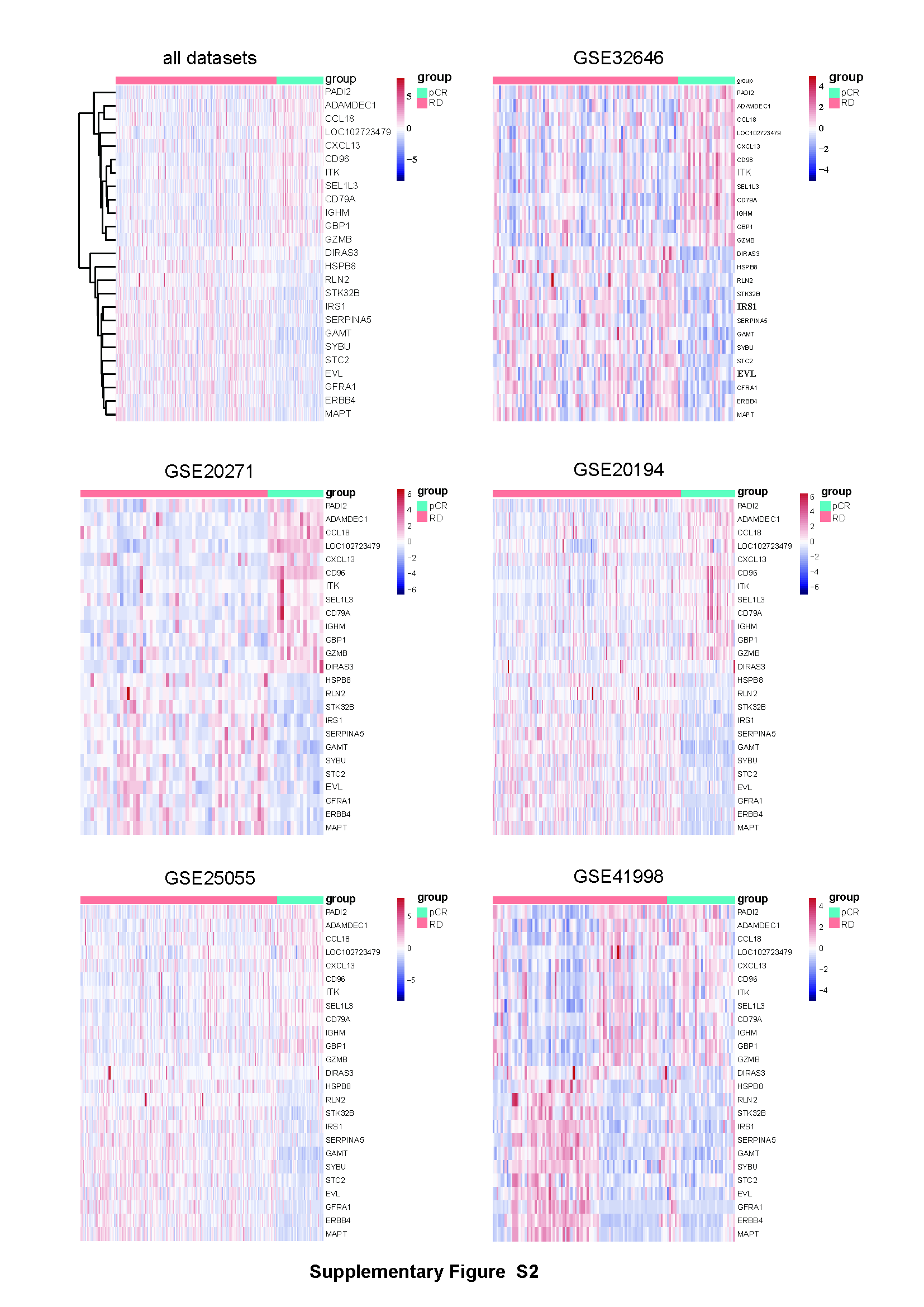

Supplement: Supplementary Figure 2 — Hierarchical clustering dendrograms of the expression patterns of 25 DEGs in all datasets. DEGs, differentially expressed genes. [file Image_2.tiff]
