# Supplementary material for: An Immune-Associated Genomic Signature Effectively Predicts Pathologic Complete Response to Neoadjuvant Paclitaxel and Anthracycline-Based Chemotherapy in Breast Cancer
Source: Front Immunol. 2021 Aug 30;12:704655. doi: 10.3389/fimmu.2021.704655 (PMC8435784; doi:10.3389/fimmu.2021.704655)
Supplement: Supplementary file 3 [file Table_1.docx]

**Table S1|** Univariate logistic regression analyses for predicting the pCR to neoadjuvant chemotherapy.

| Variable | Univariate logistic regression analyses | | |
| --- | --- | --- | --- |
|  | **β** | **OR (95% CI)** | ***P* value** |
| Age (years) |  |  |  |
| <65 | Reference |  |  |
| ≥65 | 0.854 | 0.426 (0.202-0.811) | 0.015 |
| Tumor stage |  |  |  |
| T0 | Reference |  |  |
| T1 | 1.042 | 2.833 (0.411-56.673) | 0.360 |
| T2 | 0.247 | 1.280 (0.202-24.716) | 0.823 |
| T3 | 0.511 | 1.667 (0.253-32.741) | 0.648 |
| T4 | 0.457 | 0.633 (0.089-12.767) | 0.690 |
| Lymph node status |  |  |  |
| negative | Reference |  |  |
| positive | 0.667 | 1.949 (1.234-3.173) | 0.005 |
| Histological grade |  |  |  |
| 1 | Reference |  |  |
| 2 | 0.327 | 1.386 (0.516-4.829) | 0.557 |
| 3 | 1.673 | 5.328 (2.082-18.082) | 0.002 |
| ER status |  |  |  |
| negative | Reference |  |  |
| positive | 2.173 | 0.114 (0.07-0.179) | ＜0.001 |
| PR status |  |  |  |
| negative | Reference |  |  |
| positive | 1.585 | 0.205 (0.124-0.327) | ＜0.001 |
| HER2 status |  |  |  |
| negative | Reference |  |  |
| positive | 1.032 | 2.806 (1.732-4.507) | ＜0.001 |
| Molecular subtype |  |  |  |
| Luminal (A/B) | Reference |  |  |
| HER2+(HR+) | 1.109 | 3.030 (1.123-7.394) | 0.019 |
| HER2+(HR-) | 2.959 | 19.282 (9.52-40.294) | ＜0.001 |
| TNBC | 2.204 | 9.065 (5.392-15.873) | ＜0.001 |
| Treatment course of Neoadjuvant therapy |  |  |  |
| Weekly T×12+FAC×4 | Reference |  |  |
| 3-weekly T×4+FAC×4 | 0.359 | 1.432 (0.505-3.565) | 0.463 |
| Prediction Score of 25-gene classifier | 0.38 | 1.462 (1.370-1.577) | ＜0.001 |

Abbreviations: ER, Estrogen Receptor; PR, Progesterone Receptor; HER2, Human Epidermal Growth Factor Receptor 2; OR, Odds ratio.
