# Supplementary material for: An Immune-Associated Genomic Signature Effectively Predicts Pathologic Complete Response to Neoadjuvant Paclitaxel and Anthracycline-Based Chemotherapy in Breast Cancer
Source: Front Immunol. 2021 Aug 30;12:704655. doi: 10.3389/fimmu.2021.704655 (PMC8435784; doi:10.3389/fimmu.2021.704655)
Supplement: Supplementary file 4 [file Table_2.docx]

**Table S2|** Correlations between the prediction scores and the expression values of 24 immune checkpoints.

| No | Immune Checkpoint | Correlation | *P* value |
| --- | --- | --- | --- |
| 1 | CD40LG | 0.4 | 2.2e−16 |
| 2 | CD48 | 0.39 | 2.2e−16 |
| 3 | IDO1 | 0.35 | 2.2e−16 |
| 4 | CD27 | 0.33 | 2.2e−16 |
| 5 | ICOSLG | 0.33 | 2.2e−16 |
| 6 | CTLA4 | 0.31 | 2.2e−16 |
| 7 | LAG3 | 0.31 | 2.2e−16 |
| 8 | CD86 | 0.29 | 4.5e−16 |
| 9 | ICOS | 0.28 | 2.2e−14 |
| 10 | CD28 | 0.28 | 9e−15 |
| 11 | CD70 | 0.27 | 3.3e−14 |
| 12 | LAIR1 | 0.27 | 4.3e−14 |
| 13 | PD1 /PDCD1 | 0.26 | 4.8e−13 |
| 14 | CD80 | 0.24 | 1.7e−11 |
| 15 | TNFSF4 | 0.21 | 1.1e−08 |
| 16 | CD244 | 0.2 | 7.5e−08 |
| 17 | TNFRSF25 | 0.2 | 2.4e−08 |
| 18 | PDCD1LG2 | 0.18 | 1.1e−06 |
| 19 | CD160 | 0.15 | 7.1e−05 |
| 20 | TNFSF15 | 0.083 | 0.023 |
| 21 | KIR3DL1 | 0.055 | 0.13 |
| 22 | VTCN1 | 0.037 | 0.31 |
| 23 | PDCD2 | 0.0073 | 0.84 |
| 24 | NRP1 | − 0.05 | 0.17 |
